# Identification of the Antagonistic Fungus *Diaporthe phoenicicola* Against Rhododendron Brown Spot Disease and Its Disease Control and Plant Growth-Promoting Efficacy

**DOI:** 10.3390/jof11100728

**Published:** 2025-10-10

**Authors:** Yajiao Sun, Jian Liu, Huali Li, Guangyao Zhu, Chengfen Zhu, Junjia Lu, Yunqiang Ma

**Affiliations:** 1 College of Landscape Architecture and Horticulture Science, Southwest Forestry University, Kunming 650224, China; 18087323192@126.com (Y.S.); jian927520@163.com (J.L.); 15912938064@163.com (H.L.); 18387942648@163.com (G.Z.); jisoozcf@163.com (C.Z.); 2 Key Laboratory of Forest Disaster Warning and Control of Yunnan Province, Southwest Forestry University, Kunming 650224, China; mayunqiang@swfu.edu.cn

**Keywords:** fungal antagonists, *Colletotrichum jiangxiense*, microbial identification, biological disease control, plant growth promotion

## Abstract

To explore superior biocontrol resources for Rhododendron brown spot disease, five antagonistic fungal strains exhibiting significant inhibitory activity against the pathogen responsible for RBS were isolated from healthy *Rhododendron hybridum* Ker Gawl leaves. Among them, strain DJW5-2-1 demonstrated the highest inhibition rate, reaching 63.88% against the pathogenic fungus. Based on morphological characteristics and multigene phylogenetic analysis (ITS, β-tubulin, and tef1-α), DJW5-2-1 was identified as *Diaporthe phoenicicola* (Traverso & Spessa) Udayanga, Crous & K.D. Hyde. Dual culture assays further confirmed its broad-spectrum antifungal activity, with inhibition rates ranging from 39.15% to 72.54% against various phytopathogenic fungi. Biochemical analyses revealed that DJW5-2-1 secretes multiple extracellular enzymes and exhibits plant growth-promoting traits. In both in vitro and potted plant efficacy assays, the biocontrol efficacy of strain DJW5-2-1 against RBS was 49.67% and 50.61%, respectively, indicating that strain DJW5-2-1 exhibits a certain level of control efficacy against RBS. Through pot experiments, we found that strain DJW5-2-1 could promote the growth of rhododendron seedlings and significantly increase growth indicators. Among these indicators, the growth-promoting rates of plant height and stem diameter were 15.27% and 41.27%, respectively. Moreover, DJW5-2-1 contributed to improved host resistance by elevating the activities of key defense-related enzymes, including superoxide dismutase (SOD), peroxidase (POD), catalase (CAT), and polyphenol oxidase (PPO). Taken together, these findings suggest that strain DJW5-2-1 represents a promising microbial agent for the integrated control of RBS and the development of fungal-based biofertilizers. Further investigation is warranted to assess its performance under field conditions and elucidate its underlying mechanisms of action.

## 1. Introduction

*Rhododendron simsii* Planch., a species within the rhododendron genus of the Ericaceae family, is a globally renowned ornamental plant and one of China’s top ten traditional famous flowers, often celebrated in Chinese folklore as the “Western Beauty among Flowers” [1]. In recent years, cultivation practices have intensified the incidence of fungal diseases affecting rhododendron, with brown spot disease, flower blight, leaf gall, and root rot among the most prevalent [2]. Of these, brown spot disease—also referred to as leaf spot or angular leaf spot—is the most widespread disease, primarily targeting leaves and causing necrotic lesions accompanied by blackish fruiting bodies in advanced stages, which often results in premature leaf drop. The pathogen persists through overwintering in infected leaves, thereby significantly reducing the plant’s ornamental value and overall vigor [3,4]. Plant brown spot disease is primarily caused by the genera *Cercospora* [5], *Septoria* [6], *Alternaria* [7], *Colletotrichum* [8], and *Pestalotiopsis* [9]. Currently, chemical fungicides such as carbendazim and thiophanate-methyl are widely applied to control brown spot diseases [10], while mancozeb and propiconazole have also demonstrated efficacy against certain leaf spot pathogens [11]. However, the frequent and prolonged use of these chemical agents has raised concerns regarding environmental contamination and the emergence of fungicide-resistant strains [12]. Consequently, there is an urgent demand for sustainable, eco-friendly disease management strategies. Biological control, characterized by high specificity, minimal disruption to natural enemies and beneficial microbiota, preservation of ecological balance, durable efficacy, and a lower risk of resistance development, has thus gained increasing importance as a vital component of sustainable forestry management.

Microbial pesticides have garnered considerable research interest for plant disease management due to their safety, high efficacy, and low likelihood of resistance development [13,14]. The primary microorganisms employed in biocontrol include antagonistic fungi, bacteria, and actinomycetes. Among these, plant endophytic fungi—microbes that inhabit internal plant tissues without causing visible symptoms or harm to their hosts [15]—play a significant role in enhancing plant disease resistance through mechanisms such as competition, hyperparasitism, and antibiosis. However, research on endophytic fungi associated with rhododendron remains limited. For instance, Tejesvi et al. [16] isolated and characterized endophytes from *Rhododendron vellereum* Hutch. ex Tagg, identifying antimicrobial and antioxidant compounds. Lin et al. [17] isolated two annular endophytic fungal strains from rhododendron roots, one of which produced proteases that may support host survival under stress. Similarly, Gu et al. [18] reported six *Pestalotiopsis* spp. from *Rhododendron dimitrum*, marking the first documentation of these endophytic *Pestalotiopsis spp*. from a rhododendron species in Yunnan, China. Although efforts have been made to isolate and characterize the diversity of rhododendron endophytes [19,20], studies investigating their biocontrol potential and underlying mechanisms remain scarce. The targeted screening and application of antagonistic endophytic fungi, therefore, represent a promising strategy for the management of RBS and offer valuable prospects for advancing biocontrol research in rhododendron species.

Endophytic fungi can inhibit the growth of phytopathogens by establishing competitive interactions with pathogenic fungi. For example, Abramczyk et al. reported [21] that the endophytic fungus *Diaporthe eres* Nitschke effectively suppresses postharvest pathogens of apple, including *Alternaria* spp. and *Botrytis cinerea* Pers. In addition to enhancing host resistance to both biotic and abiotic stresses, endophytic fungi are known to produce a wide array of bioactive compounds. Yehia et al. [22] demonstrated that secondary metabolites produced by *Cladosporium cladosporioides* (Fresen.) G.A. de Vries, an endophytic fungus, exhibits strong antimicrobial activity, significantly inhibiting the growth of various bacterial and phytopathogenic fungal strains. Similarly, Segaran et al. [23] isolated an endophytic fungus from *Andrographis paniculata* Wall. ex Nees that suppressed pathogenic fungi through direct hyphal interactions, including coiling around pathogen hyphae, leading to mycelial contraction and disintegration.

Endophytic fungi also exhibit notable plant growth-promoting traits, primarily through mechanisms such as phosphate solubilization, potassium solubilization, and nitrogen fixation [24], which facilitate soil mineral and nitrogen uptake as well as the secretion of plant growth hormones. For instance, Guo et al. [25] isolated a phosphate-solubilizing dark septate endophyte (DSE) from *Vaccinium corymbosum* L., the inoculation of which significantly enhanced seedling growth and phosphorus assimilation. Similarly, treatment of potato plants with fermentation broth from the endophytic fungus SD1-4 led to a marked reduction in leaf spot disease severity alongside improvements in plant height, root length, and biomass compared to untreated controls [26]. Endophytic antagonists suppress fungal pathogens by secreting proteinaceous antimicrobial enzymes—such as cellulase, protease, and glucanase—and siderophores that competitively limit pathogen iron acquisition [27]. Li et al. [28] identified a *Neocosmospora solani* (Mart.) L. Lombard & Crous strain that produces cellulase, glucanase, protease, and siderophores, with enzyme secretion levels positively correlating with disease suppression efficacy. As long-term plant symbionts, endophytic fungi secrete bioactive metabolites through fermentation that enhance host stress tolerance by modulating defense-related enzyme systems. For example, Li et al. [29] demonstrated that co-inoculation with arbuscular mycorrhizal fungi (AMF) and endophytic fungi significantly increased antioxidant enzyme activities in navel orange leaves, thereby activating the trees’ antioxidative defense. Zou et al. [30] reported that root inoculation with the DSE *Falciphora oryzae* C.L. Zhang rapidly colonized pepper roots and upregulated POD, CAT, and ascorbate peroxidase (APX) activities, enhancing salt stress tolerance. Despite these advances, studies on the inhibitory effects of endophytic fungi against RBS and their growth-promoting mechanisms remain limited, with underlying molecular pathways yet to be elucidated.

This study investigates *Colletotrichum jiangxiense* F. Liu & L. Cai strain XY-4, identified as the causal agent of RBS, and focuses on the isolation and characterization of an antagonistic strain, DJW5-2-1, from healthy rhododendron plants. Strain identification was conducted through a combination of single-colony morphological observations and multilocus sequence analysis targeting conserved fungal genes. Comprehensive assessments were performed to evaluate the biocontrol and growth-promoting potential of DJW5-2-1, including its antifungal activity against multiple phytopathogenic fungi, secretion of extracellular hydrolases, pot-based plant growth promotion trials, and its effect on host defense enzyme activities. This work aims to provide a promising microbial resource for the biological control of brown spot disease and to establish a theoretical basis for future research into the biocontrol mechanisms of beneficial microorganisms.

## 2. Materials and Methods

### 2.1. Plant Materials Tested

In September 2023, healthy *R. hybridum* Ker Gawl leaves were collected from the Yuxi *Rhododendron* Garden at Kunming Botanical Garden (E102°44′15.2″, N25°07′04.9″) and promptly transported to the laboratory for endophytic fungal isolation.

### 2.2. Test Strains and Culture Media

In our preliminary experiments, the pathogenic fungus XY-4 (*C. jiangxiense*), responsible for RBS, was isolated. Additional pathogenic strains isolated and preserved by our research group from diseased tissues in related studies include C4 (*Apiospora intestini* Kajale, Sonawane & Roh. Sharma), the causal agent of ivy leaf blight; FX-4 (*Alternaria tenuissima* (Kunze) Wiltshire), causing *Impatiens hawkeri* W.Bull leaf spot; Q13 (*C*. *aotearoa* B. Weir & P.R. Johnst.), responsible for *Begonia* L. anthracnose; MFR-4 (*Fusarium sambucinum* Fuckel), causing *Hibiscus mutabilis* L. leaf spot; GB1 (*Botrytis cinerea*), the agent of *Vaccinium corymbosum* L. gray mold; X64 (*Epicoccum sorghinum* (Sacc.) Aveskamp, Gruyter & Verkley), causing *Hydrangea* L. leaf spot; and Y1 (*Epicoccum nigrum* Link), associated with *Rhus chinensis* Mill. leaf spot. The relevant medium formulations are detailed in Table S1 of the Supporting Information.

### 2.3. Screening of Biocontrol Fungi and Determination of Their Broad-Spectrum Antifungal Activity

Fresh healthy rhododendron leaves were collected, rinsed with sterile water, and aseptically cut into small segments using a sterile scalpel within a laminar flow hood. Surface sterilization was performed sequentially by immersing the leaf segments in 75% ethanol for 2 min, followed by three rinses with sterile water; subsequently, the segments were immersed in 1% sodium hypochlorite for 30 s, followed by another three sterile water rinses. After sterilization, the leaf segments were blotted dry on sterile filter paper and placed onto potato dextrose agar (PDA) plates for incubation at 28 °C for 3–7 days. Emerging colonies surrounding the leaf segments were monitored daily, isolated, and subcultured onto fresh PDA plates for purification through three consecutive passages. To confirm the efficacy of surface sterilization, the final rinse water was streaked onto PDA plates as a control [31]. Antifungal activity was assessed using the five-point dual culture assay with reference to Marwa’s method [32]. Mycelial discs (5 mm in diameter) of both the pathogenic fungi and endophytic isolates were obtained using a sterile cork borer (Solarbio, Beijing, China). The pathogenic disc was positioned at the center of a PDA plate, while four endophytic discs were inoculated equidistantly at 2 cm from the center. Plates inoculated solely with the pathogenic disc served as controls. All plates were incubated at 28 °C, and inhibition rates were calculated after 7 days. Each treatment was performed in triplicate.

The strain exhibiting the strongest antifungal activity was selected to evaluate its broad-spectrum efficacy against a range of phytopathogens, including C4 (causal agent of ivy leaf blight), FX-4 (*Impatiens hawkeri* leaf spot), Q13 (*Begonia anthracnose*), MFR-4 (*Hibiscus mutabilis* leaf spot), GB1 (*Vaccinium corymbosum* gray mold), X64 (*Hydrangea* leaf spot), and Y1 (*Rhus chinensis* leaf spot). The calculation formula is as follows:

Inhibition Rate (%) = [(Control Pathogen Colony Diameter − Treatment Pathogen Colony Diameter)/Control Pathogen Colony Diameter] × 100%

### 2.4. Identification and Phylogenetic Tree Analysis of Biocontrol Fungi

The purified biocontrol strain was inoculated onto PDA, potato sucrose agar (PSA), and corn agar (CA) media and incubated at 28 °C for 7 days. Colony morphology, size, and color were observed, documented, and photographed.

Following the method described by Su et al. [33] with slight modifications, the slide culture technique was employed. A thin layer (~1 mm) of a mixed medium (Czapek medium supplemented with alfalfa juice) was poured into Petri dishes and subsequently cut into 1.5 cm × 1.5 cm cubes using a sterile scalpel. Biocontrol fungal mycelia were inoculated onto the edges of these cubes, which were then covered with cover slips. Cultures were incubated at 28 °C under a 12 h light/12 h dark photoperiod for 3–7 days. Spore morphology was examined and photographed using a biological microscope (Olympus CX33, Tokyo, Japan).

Genomic DNA of the strain was extracted using a fungal DNA extraction kit following the modified CTAB method [34]. PCR amplification targeted the internal transcribed spacer (ITS) region [35], translation elongation factor 1-alpha (TEF1-α) [36], and β-tubulin (TUB2) gene [37] sequences. The ITS amplification mixture contained 12.5 μL PCR mix, 9.5 μL double-distilled water, 1 μL each of primers ITS1 and ITS4, and 1 μL DNA template. The PCR cycling conditions were initial denaturation at 95 °C for 5 min; 35 cycles of 94 °C for 30 s, annealing at 52 °C for 45 s, and extension at 72 °C for 50 s; followed by a final extension at 72 °C for 10 min, then storage at 4 °C. For TEF1-α and TUB2 amplifications, the reaction mixture comprised 15 μL PCR mix, 12 μL double-distilled water, 1 μL each of forward and reverse primers, and 1.2 μL DNA template. The TUB2 PCR program included initial denaturation at 94 °C for 5 min; 30 cycles of 94 °C for 30 s, annealing at 55 °C for 45 s, and extension at 72 °C for 1 min; with a final extension at 72 °C for 10 min, and preservation at 4 °C. The TEF1-α program was as follows: initial denaturation at 94 °C for 2 min; 31 cycles of 94 °C for 30 s, annealing at 55 °C for 30 s, and extension at 72 °C for 30 s; concluding with a final extension at 72 °C for 2 min, and storage at 4 °C. PCR products were sequenced by Beijing Qingke Biotech Co., Ltd. (Beijing, China). The resulting sequences were aligned using BLAST on the NCBI database (https://www.ncbi.nlm.nih.gov/, accessed 5 June 2025). Reference sequences were selected according to relevant literature. Phylogenetic analysis was conducted using the neighbor-joining method in MEGA11 (Molecular Evolutionary Genetics Analysis Version 11) software, with bootstrap values set at 1000 replicates to assess the robustness of the tree, thereby enabling species identification of the biocontrol strain.

### 2.5. Determination of Extracellular Hydrolases in Biocontrol Fungi

Cellulase, protease, and amylase activities were assayed following the protocols described by Sopalun, Mwendwa et al. [38,39]. β-Glucanase activity was determined according to the method of El-Shora et al. [40], while chitinase activity was evaluated using the procedure outlined by Malik et al. [41]. The strains were inoculated onto specific enzyme-indicating media and incubated at 28 °C for 5 days. The formation of clear zones or halos surrounding the colonies was used as an indicator of enzyme production.

### 2.6. Biocontrol Efficacy of Biocontrol Fungi

In vitro biocontrol efficacy: A spore suspension of the fungus with a concentration of 1 × 10^7^ CFU/mL was prepared using sterile water. Rhododendron leaves were sterilized and punctured, after which the pathogen spore suspension was dropped onto the wound sites, with 1 drop per wound and 30 μL per drop. After 24 h, the spore suspension of biocontrol strain DJW5-2-1 was dropped onto the punctured wound sites of rhododendron leaves, with the treatment of dropping clear water set as the control. Each treatment had 3 replicates. After incubation at 28 °C with moisture retention for 7 days, the lesion size was measured, and the biocontrol efficacy and disease index were calculated. The disease grading criteria referred to the method described by Sun et al. [42]. The calculation formula is as follows:

Disease index = [Σ (number of diseased leaves at each grade × value of the corresponding grade)]/[total number of leaves surveyed × highest grade value].

Biocontrol efficacy (%) = (disease index of the control group − disease index of the treatment group)/disease index of the control group × 100%.

Potted plant biocontrol efficacy: The experiment was conducted in a greenhouse maintaining day/night temperatures of approximately 30 °C/23 °C. One-year-old rhododendron seedlings were transplanted individually into flowerpots (18 cm × 20 cm) containing 3000 g of sterilized soil, with six pots per treatment. Spore suspensions of DJW5-2-1 (1 × 10^7^ CFU/mL) and pathogen XY-4 (1 × 10^7^ CFU/mL) were prepared according to the method described above. The spray inoculation method was employed: approximately 20 mL of XY-4 spore suspension was sprayed first, and 24 h later, 20 mL of DJW5-2-1 spore suspension was sprayed. Spraying with sterile water served as the control, with 3 replicates for each treatment. Seven days later, the biocontrol efficacy and disease index were calculated using the same method as described above.

### 2.7. Study on Growth-Promoting Characteristics of Biocontrol Fungi

#### 2.7.1. Determination of Growth-Promoting Ability of Biocontrol Fungi

Media for detecting nitrogen fixation, phosphate solubilization, and potassium solubilization were prepared following the method described by Wang et al. [43]. The medium for siderophore production was obtained commercially as CAS detection medium (10.87 g dissolved in water, adjusted to a final volume of 1000 mL). The strain was inoculated onto nitrogen-fixing medium and incubated at 28 °C for 5 days, with continuous subculturing performed three times. The strain was considered nitrogen-fixing if it maintained growth after the third subculture; otherwise, it was deemed negative. For phosphate solubilization, potassium solubilization, and siderophore production assays, the strain was inoculated onto the respective media and incubated at 28 °C for 5 days. The formation of clear zones or halos around the colonies indicated positive growth-promoting activity, whereas their absence indicated a lack of such activity.

The production of IAA was assessed qualitatively and quantitatively according to the method of Abdelhamid et al. [44]. Compared to the control, a deeper color intensity corresponded to a higher level of IAA production, whereas a lighter color indicated lower production.

#### 2.7.2. Pot Experiment on Growth Promotion by Biocontrol Fungi

The growth status of experimental materials and the number of pots per treatment were consistent with those in Section 2.6. Each pot was irrigated with 50 mL of an endophytic fungal spore suspension (1 × 10^7^ cfu/mL), while the control group received an equivalent volume of sterile water. Treatments were replicated three times, and irrigation was applied every 5 days. After 45 days, growth parameters—including seedling height, stem diameter, leaf area, fresh weight, dry weight, and chlorophyll content—were measured for each treatment. Leaf area was analyzed using ImageJ 1.54g software, and the determination of chlorophyll content was performed via the acetone method, following the protocol described by Hartmut et al. [45].

### 2.8. Effect of Biocontrol Fungi on Defense Enzyme Activities in Rhododendron Seedlings

The planting conditions for rhododendron seedlings were consistent with those used in the growth-promotion experiment, with six pots per treatment and three replicates per treatment. Endophytic fungal spore suspension and pathogenic fungal spore suspension were each prepared at a concentration of 1 × 10^7^ cfu/mL, following the methods described above. Four treatment groups were established as follows:

Treatment 1: Each pot was irrigated with 50 mL of endophytic fungal spore suspension via root drenching.

Treatment 2: Each pot was irrigated with 50 mL of pathogenic fungal spore suspension via root drenching.

Treatment 3: Each pot was irrigated first with 25 mL of pathogenic fungal spore suspension, followed 24 h later by 25 mL of endophytic fungal spore suspension via root drenching.

Treatment 4: Each pot was irrigated with 50 mL of sterile water via root drenching (control).

Root drenching was conducted every 7 days. Following the second drenching, leaves from the same position were sampled on days 1, 3, 5, 7, 9, and 11 to assess defense enzyme activities. SOD activity was determined using the nitroblue tetrazolium (NBT) photoreduction assay, while PPO activity was measured via the catechol method. Detailed protocols were adapted from Wu et al. and Shen et al. [46,47].

### 2.9. Statistical Analysis of Data

Experimental data were analyzed by analysis of variance (ANOVA) using GraphPad Prism 9.5 software, and significant differences among means were determined by Duncan’s multiple range test; differences were considered significant at *p* < 0.05.

## 3. Results and Analysis

### 3.1. Isolation and Screening of Biocontrol Fungi

**Figure 1:**
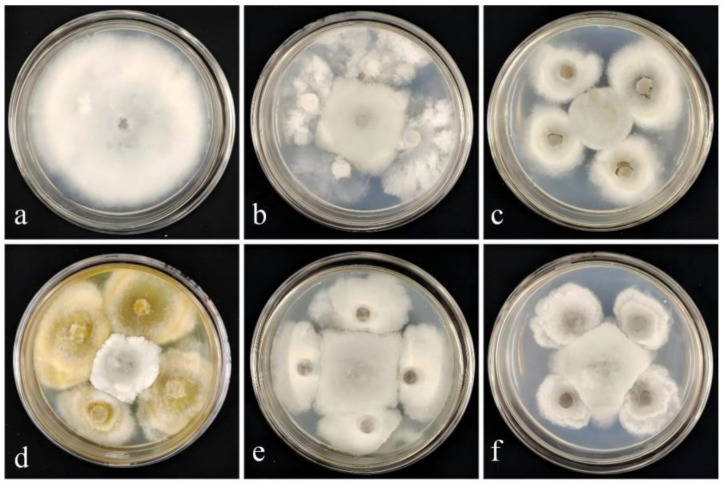
Inhibitory effect of strain DJW5-2-1 against the pathogenic fungus causing RBS. (**a**) Control group, inoculated solely with pathogenic fungus XY-4; (**b**) Inhibitory effect of strain DJY1-1; (**c**) Inhibitory effect of strain DJW5-2; (**d**) Inhibitory effect of strain DJW5-2-1; (**e**) Inhibitory effect of strain DJW5-3; (**f**) Inhibitory effect of strain DJW5-4.

In total, 21 endophytic fungal strains were isolated in this study. Based on the results of plate antagonism assays, five strains exhibiting biocontrol potential were selected (Figure 1). Each of these five strains demonstrated an inhibition rate exceeding 50% against the pathogenic fungus XY-4, the causal agent of RBS (Table 1). Among them, strain DJW5-2-1 exhibited the strongest antagonistic activity, with an inhibition rate of 63.88%. Consequently, DJW5-2-1 was chosen for further investigation in subsequent experiments.

### 3.2. Effect of Strain DJW5-2-1 on Various Pathogenic Fungi

**Figure 2:**
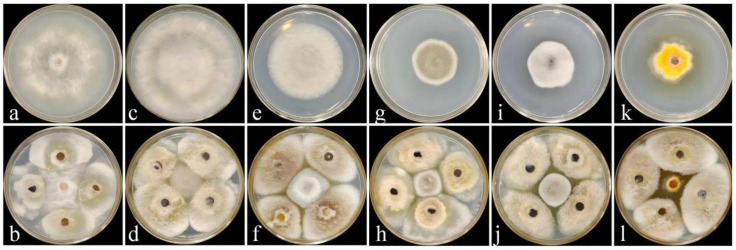
Inhibitory effect of strain DJW5-2-1 against 6 pathogenic fungi. (**a**,**b**) Pathogen C4 causing *Hedera helix* leaf blight in the control group and its corresponding treatment group, respectively. (**c**,**d**) Pathogen GB1 causing gray mold of *Vaccinium corymbosum* in the control group and its corresponding treatment group, respectively. (**e**,**f**) Pathogen Q13 causing anthracnose of *Begonia semperflorens* in the control group and its corresponding treatment group, respectively. (**g**,**h**) Pathogen FX-4 causing leaf spot of *Impatiens hawkeri* in the control group and its corresponding treatment group, respectively. (**i**,**j**) Pathogen X64 causing leaf spot of *Hydrangea macrophylla* in the control group and its corresponding treatment group, respectively. (**k**,**l**) Pathogen Y1 causing leaf spot of *Rhus chinensis* in the control group and its corresponding treatment group, respectively.

**Figure 3:**
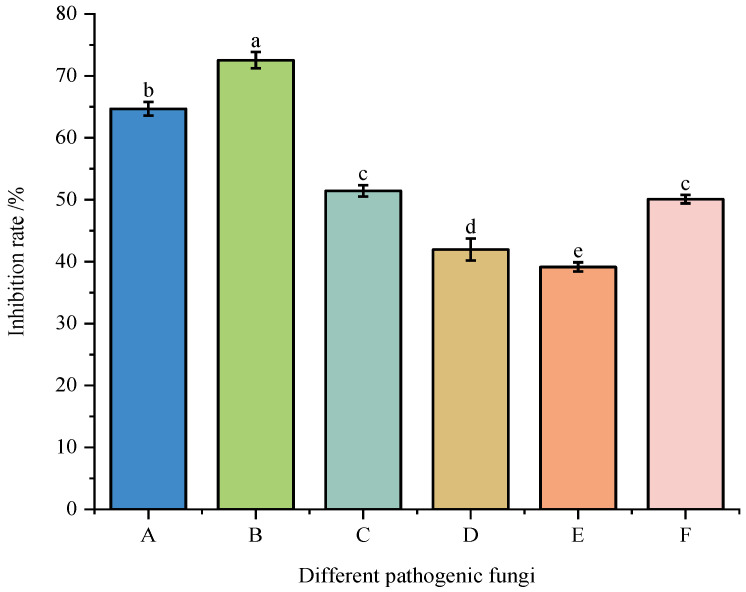
Inhibition rates of strain DJW5-2-1 against six pathogenic fungi. (A–F) represent C4, GB1, Q13, FX-4, X64, and Y1, respectively. Note: Different lowercase letters indicate significant differences at *p* < 0.05.

The antagonistic activity of strain DJW5-2-1 against six plant pathogenic fungi is presented in Figure 2 and Figure 3. The results showed that DJW5-2-1 significantly inhibited the mycelial growth of all tested pathogens, with inhibition rates in descending order as follows: *Vaccinium corymbosum* gray mold (72.54%) > *Hedera helix* leaf blight (64.69%) > anthracnose of *Begonia semperflorens* (51.43%) > leaf spot of *Rhus chinensis* (50.09%) > leaf spot of *Impatiens hawkeri* (41.96%) > leaf spot of *Hydrangea macrophylla* (39.15%). These results indicate that DJW5-2-1 possesses a broad-spectrum antifungal capacity and exhibits strong inhibitory effects against a variety of common phytopathogenic fungi.

### 3.3. Identification of Strain DJW5-2-1

#### 3.3.1. Morphological Identification

**Figure 4:**
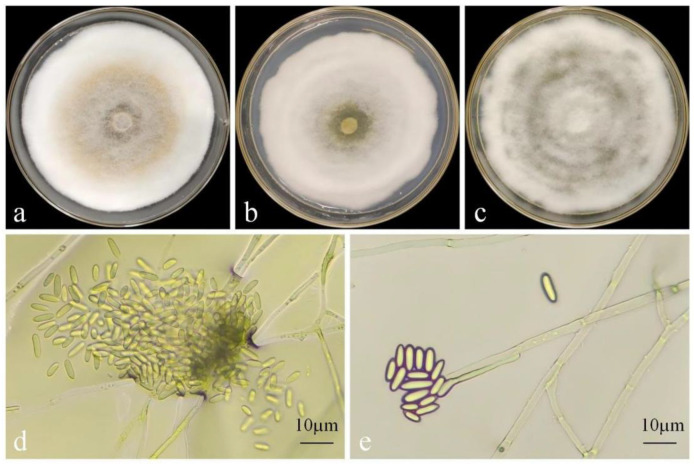
Morphological characteristics of strain DJW5-2-1. (**a**) Colony grown on PDA medium for 7 days; (**b**) colony grown on PSA medium for 7 days; (**c**) colony grown on CA medium for 7 days; (**d**) conidia; and (**e**) mycelia and conidiophores.

After incubation on PDA medium at 28 °C in the dark for 7 days, strain DJW5-2-1 formed colonies with a diameter of 85 mm, exhibiting a regular circular shape, smooth margins, and yellow pigmentation. Under the same conditions on PSA medium, the colony diameter measured 77.01 mm and was characterized by dense white mycelium. On CA medium, the mycelium completely covered the Petri dish, producing white colonies with concentric rings radiating outward from the center. Microscopic observation revealed α-conidia that were elliptical, unicellular, hyaline, and blunt at both ends, measuring 6.95–9.08 × 2.33–3.19 µm (mean: 8.02 × 2.76 µm, n = 30) (Figure 4). β-conidia were not detected. Based on these morphological features and comparison with the descriptions reported by Cha et al. [48], strain DJW5-2-1 was preliminarily identified as belonging to the genus *Diaporthe*.

#### 3.3.2. Phylogenetic Tree Construction

**Figure 5:**
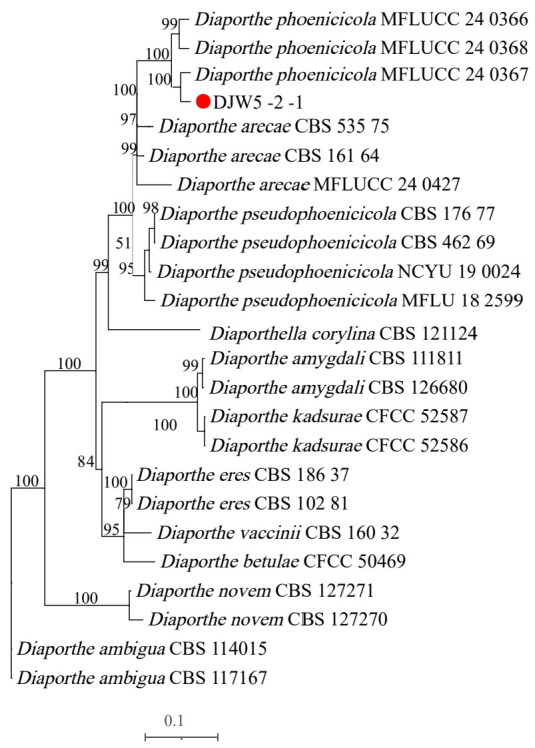
Multi-locus phylogenetic tree of strain DJW5-2-1.

PCR amplification of strain DJW5-2-1 using ITS, TEF, and TUB2 primers generated gene fragments of 702 bp, 383 bp, and 808 bp, respectively. The resulting sequences were submitted to the GenBank database. Following BLAST alignment, homologous reference sequences were selected and downloaded (Table 2). These sequences were concatenated in the order ITS–TEF–TUB2 to construct a multilocus phylogenetic tree. As shown in Figure 5, strain DJW5-2-1 clustered within the same clade as *Diaporthe phoenicicola* MFLUCC:24-0367, with a bootstrap support value of 100%. The morphological characteristics were consistent with the molecular identification results, confirming that strain DJW5-2-1 belongs to *D. phoenicicola*.

### 3.4. Determination of Cell Wall-Degrading Enzymes Produced by Strain DJW5-2-1

**Figure 6:**
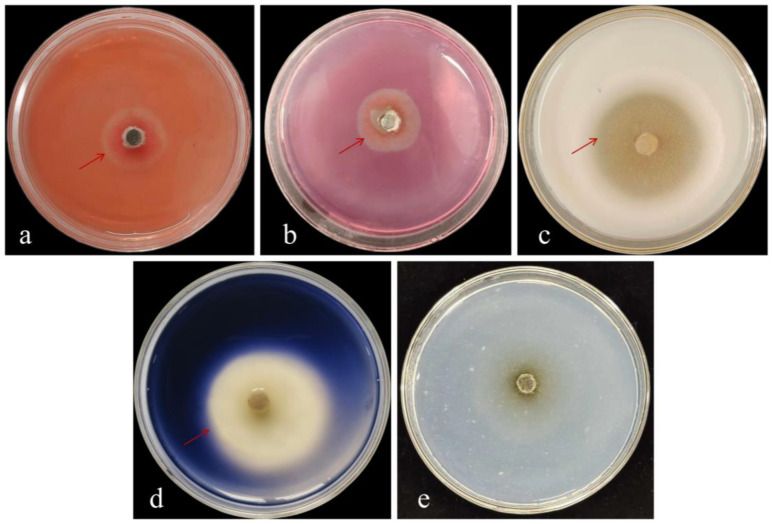
Determination of cell wall-degrading enzyme activities of strain DJW5-2-1. (**a**) β-glucanase; (**b**) cellulase; (**c**) protease; (**d**) amylase; (**e**) chitinase. The red arrow indicates the location of the hydrolysis zone.

The cell wall-degrading enzyme activity of strain DJW5-2-1 is presented in Figure 6. Clear hydrolysis zones were observed around the colonies on glucanase, cellulase, protease, and amylase detection plates, indicating the production of these extracellular enzymes by DJW5-2-1. Each enzyme specifically degraded its corresponding substrate—glucan, sodium carboxymethyl cellulose, protein, and starch—in the medium, resulting in the formation of transparent zones. Although DJW5-2-1 was able to grow on the chitinase detection medium, no clear zone was observed, suggesting that it does not secrete chitinase. Overall, these results demonstrate that strain DJW5-2-1 produces multiple extracellular hydrolases, which contribute to its strong biocontrol potential.

### 3.5. Biocontrol Efficacy of Strain DJW5-2-1

**Figure 7:**
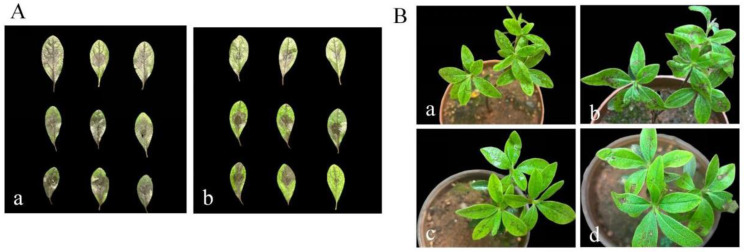
Biocontrol efficacy of strain DJW5-2-1 on in vitro leaves and in vivo potted plants. (**A**) Biocontrol on in vitro leaves: (**a**) control group; (**b**) treatment group; (**B**) biocontrol efficacy of in vivo potted plants: (**a**) control group at 0 d; (**b**) control group at 7 d; (**c**) treatment group at 0 d; (**d**) treatment group at 7 d.

As shown in Figure 7 and Table 3, in the in vitro biocontrol assay, 7 days after spraying the DJW5-2-1 spore suspension, the disease index of the control group was 79.63, whereas the rhododendron leaves treated with biocontrol strain DJW5-2-1 exhibited relatively mild disease symptoms, with a disease index of 40.37 and a biocontrol efficacy of 49.67%. Results from the potted plant biocontrol assay demonstrated that 7 days after spraying the biocontrol strain spore suspension (24 h after spraying the pathogen spore suspension), the disease index was 18.33, and the biocontrol efficacy reached 50.61%. These findings indicate that strain DJW5-2-1 exhibits a certain level of biocontrol efficacy against RBS caused by *C. jiangxiense*.

### 3.6. Plant Growth-Promoting Ability of Strain DJW5-2-1

#### 3.6.1. Determination of Plant Growth-Promoting Functions of Strain DJW5-2-1

**Figure 8:**
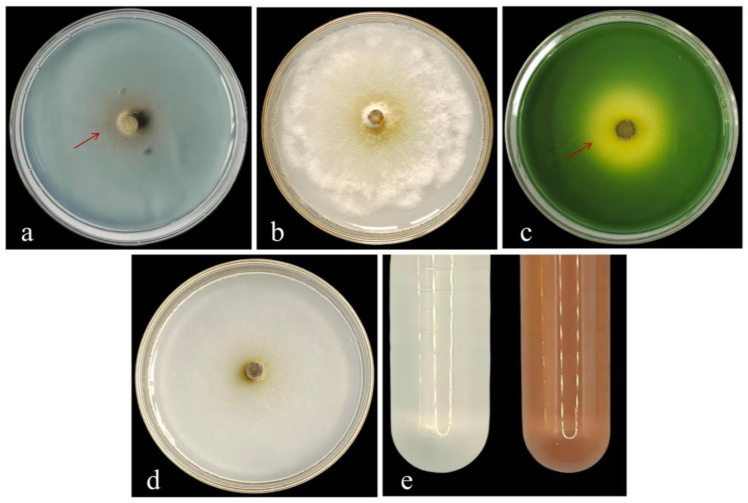
Determination of plant growth-promoting abilities of strain DJW5-2-1. (**a**) Siderophore production; (**b**) phosphate-solubilizing ability; (**c**) potassium-releasing ability; (**d**) nitrogen-fixing ability; (**e**) IAA-producing ability (left: control; right: treatment). The red arrow indicates the location of the hydrolysis zone.

The plant growth-promoting traits of strain DJW5-2-1 are summarized in Figure 8. The strain grew on CAS medium, producing distinct orange halos indicative of siderophore secretion. Although DJW5-2-1 exhibited robust growth on organophosphorus medium, it did not form transparent zones, suggesting a lack of phosphate-solubilizing ability. On modified Aleksandrov medium, the formation of orange-yellow halos indicated the strain’s potassium-solubilizing capacity. Furthermore, DJW5-2-1 sustained growth after five successive subcultures on Ashby medium, demonstrating its nitrogen-fixing capability.

Qualitative analysis confirmed that strain DJW5-2-1 produces IAA, as evidenced by a deeper color development compared to the control, indicating a strong IAA synthesis capacity. Using the method described by Abdelhamid, a standard curve was established with the following equation:

y = 0.0268x + 0.13 (R^2^ = 0.9926)

Based on this calibration, the IAA concentration produced by DJW5-2-1 was calculated to be 9.09 mg/L.

#### 3.6.2. Growth-Promoting Effect of Strain DJW5-2-1 on Rhododendron Seedlings

**Figure 9:**
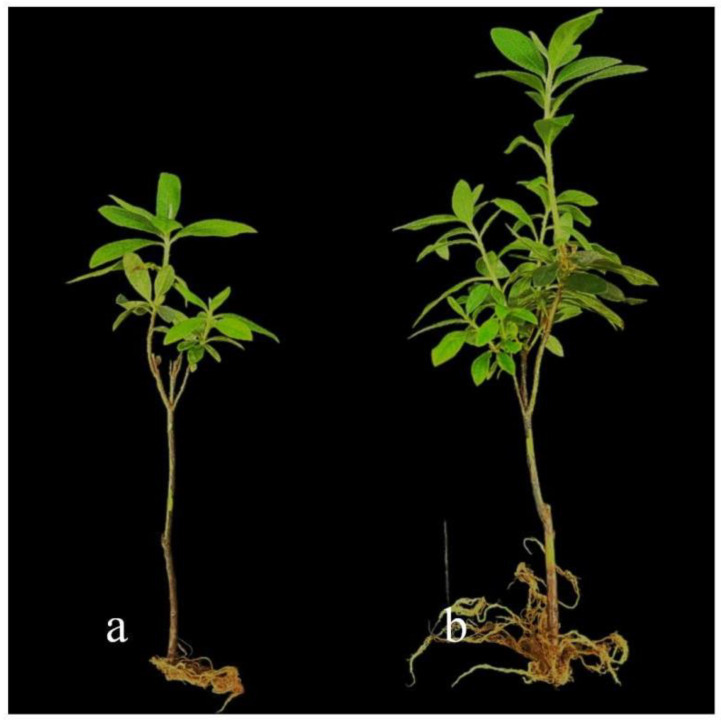
Growth-promoting effect of strain DJW5-2-1 on rhododendron seedlings. (**a**) Control group; (**b**) treatment group.

The growth-promoting effects of strain DJW5-2-1 on rhododendron seedlings are illustrated in Figure 9. Plants treated with DJW5-2-1 exhibited significantly greater height compared to the control. As summarized in Table 4, the average growth parameters for treated seedlings were plant height 23.4 cm, root length 5.62 cm, stem diameter 0.34 cm, leaf area 3.16 cm^2^, chlorophyll content 3.44 mg/g, aboveground fresh weight 2.37 g, aboveground dry weight 1.11 g, underground fresh weight 1.15 g, and underground dry weight 0.40 g. Compared to controls, these values corresponded to growth-promoting increases of 15.27%, 13.53%, 41.27%, 34.47%, 55.66%, 82.31%, 94.73%, 259.37%, and 185.71%, respectively. Compared with the control group, its fresh weight and dry weight increased significantly. This fungus has growth-promoting abilities (e.g., IAA production, siderophore production, potassium solubilization) and thus promotes rhododendron root development. Additionally, it may improve rhizosphere soil structure and enhance soil water and nutrient retention, creating a stable root growth environment and indirectly boosting root fresh and dry weights.

### 3.7. Induced Resistance of Strain DJW5-2-1 in Rhododendron Seedlings

**Figure 10:**
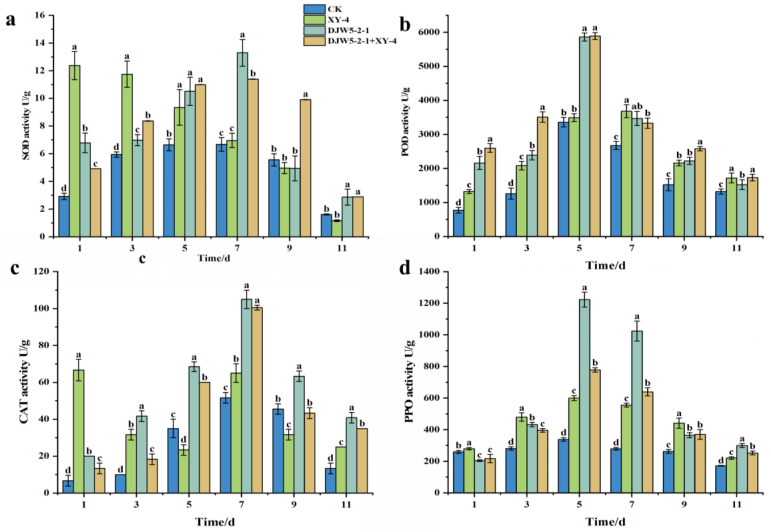
Effects of strain DJW5-2-1 on defense enzyme activities in rhododendron seedlings. (**a**) Effect on SOD activity; (**b**) effect on POD activity; (**c**) effect on CAT activity; (**d**) effect on PPO activity. Note: Different lowercase letters indicate significant differences at *p* < 0.05.

The activities of defense-related enzymes in rhododendron leaves were measured at 1, 3, 5, 7, 9, and 11 days post-inoculation (Figure 10). Compared to the control group treated with sterile water (CK), inoculation with strain DJW5-2-1 significantly increased the activities of SOD, POD, CAT, and PPO. SOD and CAT activities peaked on day 7, reaching 1.37- and 2.03-fold of the control, respectively, while POD and PPO activities peaked earlier on day 5, with increases of 1.73- and 3.6-fold, respectively. These findings suggest that DJW5-2-1 inoculation enhances defense enzyme activities in rhododendron, thereby inducing systemic resistance against pathogens.

In the group where endophytic fungi were inoculated 24 h after pathogen inoculation, the activities of SOD, POD, CAT, and PPO exhibited a pattern of initial increase followed by a decline, peaking between days 5 and 7 at 1.18-, 1.79-, 1.95-, and 2.30-fold higher than the control, respectively. These results indicate that, under pathogen-induced stress, inoculation with strain DJW5-2-1 can mitigate the decline in defense enzyme activities, thereby enhancing the plant’s disease resistance.

In the group inoculated solely with pathogen XY-4, SOD activity steadily declined from day 1 to day 11, with a rapid decrease between days 1 and 5 followed by a slower decline from days 7 to 11. POD activity initially increased before declining but remained consistently higher than the control. CAT activity decreased from day 1 to day 5, then showed a subsequent increase. PPO activity gradually increased throughout the 11-day period, maintaining levels above those of the control. These patterns suggest that the pathogen induces a moderate activation of the rhododendron defense system.

## 4. Discussion

*Diaporthe phoenicicola* belongs to the class Sordariomycetes. The genus *Diaporthe* encompasses a large number of species exhibiting diverse ecological roles, including pathogenicity, endophytism, and saprotrophy. Members of this genus possess a broad host range and have a global distribution [49]. Traditionally recognized primarily as plant pathogens, recent studies have revealed their endophytic capabilities, thereby broadening our understanding of their ecological functions. For instance, multigene phylogenetic analysis of *Diaporthe* species isolated from *Citrus grandis* cv. Tomentosa in China identified 11 endophytic species, including two novel taxa (*D. endocitricola* and *D. guangdongensis* Z.Y. Dong, M. Luo, M.M. Xiang, K.D. Hyde), confirming the genus’s endophytic colonization in economically important crops [50]. Similarly, Lambert et al. [51] described four new endophytic species (*D. brideliae*, *D. cameroonensis*, *D. pseudoanacardii*, and *D. rauvolfiae* L. Schweizer, C. Lamb. & Y. Marín) isolated from plants in Cameroon, highlighting *Diaporthe* as a dominant fungal population in the region’s flora. Additionally, Toghueo et al. [52] demonstrated that strain EB4 of *Diaporthe*, isolated from the salt-tolerant forage grass *Festuca rubra* L., significantly enhanced salt tolerance, promoted growth, and regulated hormonal balance in triticale and perennial ryegrass. Furthermore, Abramczyk et al. [21] reported that *D. eres* strain 1420S, isolated from Prunus species, inhibited several plant pathogens—including *Verticillium dahliae* Klebahn and *Botrytis cinerea*—with inhibition rates ranging from 20% to 40%. Collectively, these findings underscore that *Diaporthe* species are widely distributed endophytic fungi capable of colonizing diverse host plants without inducing disease.

Previous studies have demonstrated that certain *Diaporthe* species can suppress pathogenic fungi by competing for resources such as nutrients and space, thereby inhibiting pathogen growth. For instance, Niaz et al. [53] isolated seven azacycloketone compounds from the mangrove endophytic fungus *Diaporthe perseae* (Zerova) R.R. Gomes, Glienke & Crous, among which chlorinated isochromophilone G (compound **1**) showed potent antimicrobial activity against *Staphylococcus aureus* Rosenbach and *Escherichia coli* Migula. Yan et al. [54] investigated the inhibition of pathogenic fungi using volatile compounds from 4 Diaporthe species, and the results showed that these compounds exerted a certain inhibitory effect on the mycelial growth of *Botrytis cinerea* and *Alternaria alternata*. Additionally, Kapoor et al. [55] found that *Diaporthe* sp. DG-S4, isolated from *Dysoxylum gotadhora* (Buch.-Ham.) Mabb. inhibited *Verticillium dahliae* growth by 70%, with scanning electron microscopy (SEM) revealing entanglement and degradation of pathogenic hyphae. Consistent with these findings, the present study identified a *Diaporthe* strain from rhododendron leaves capable of inhibiting the growth of multiple phytopathogens, further confirming the antimicrobial potential of *Diaporthe* fungi in plant disease management.

Furthermore, several studies have reported that certain endophytic fungi secrete a variety of bioactive enzymes, such as chitinases, glucanases, and cellulases, which directly inhibit pathogenic fungi. Chamier et al. [56] demonstrated that *Diaporthe* species produce cell wall-degrading enzymes, including chitinases, cellulases, and proteases. Notably, chitinases are considered crucial for antifungal activity, while cellulases are implicated in host infectivity. Proteases, on the other hand, contribute to degrading host defense proteins, facilitating pathogen colonization. Consistent with these findings, the present study found that cellulase, β-glucanase, protease, and amylase secreted by strain DJW5-2-1 may contribute to the degradation of pathogenic fungi’s cell walls and the suppression of their growth. In the study by Yuan et al. [57], four endophytic fungal strains were isolated from cotton and used to control cotton Verticillium wilt. The results demonstrated that these strains could significantly reduce the disease incidence and disease index of cotton Verticillium wilt, with biocontrol efficacy ranging from 26% to 67% 25 days after inoculation. This is consistent with the results of the present study.

Endophytic fungi can also indirectly enhance plant disease tolerance by improving nutritional status and promoting growth. For example, Toghueo et al. [52] reported that the *Diaporthe* strain EB4, isolated from wild grasses, synthesizes IAA in vitro and elevates endogenous levels of IAA and gibberellin (GA) in host plants, thereby stimulating root and shoot biomass accumulation. Similarly, da Silva et al. [58] found that *D. masirevicii* R.G. Shivas, L. Morin, S.M. Thomps. & Y.P. Tan strain JB270 promotes tomato taproot elongation, increases lateral root density, and enhances leaf chlorophyll content along with the accumulation of nitrogen, phosphorus, and potassium. Castelli et al. [59] identified *Diaporthe* strains capable of simultaneously solubilizing phosphate, producing IAA, and secreting chitinases, suggesting their potential as multifunctional biofertilizers. The combined phosphate- and potassium-solubilizing abilities of these strains significantly improve soil nutrient availability, thereby promoting plant growth. In the related study by Li et al. [28], a strain of antagonistic fungus CY12 was isolated from *Quercus spinosa* David. This strain possesses the abilities of potassium-solubilizing, nitrogen-fixing, and IAA-producing, and can promote the growth of impatiens seedlings. In the present study, strain DJW5-2-1 demonstrated potassium solubilization, nitrogen fixation, siderophore production, and a robust capacity for IAA synthesis, with an IAA yield of 9.09 mg/L. In pot experiments, plants treated with the DJW5-2-1 fermentation broth showed significant increases in plant height, root length, stem diameter, leaf area, chlorophyll content, and biomass (both fresh and dry weights of aboveground and belowground tissues) compared to controls. These findings indicate that strain DJW5-2-1 strongly promotes plant growth, thereby enhancing overall plant health and resistance to pathogen infection. This may be attributed to the fact that this strain is capable of secreting multiple plant growth-promoting substances, thereby providing nutrients for the growth of rhododendron seedlings.

Endophytic fungi can modulate the activity of antioxidant enzymes in plants, thereby enhancing physiological stability under pathogen-induced stress. In this study, rhododendron seedlings treated with pathogens, endophytic fungi, or their combination exhibited antioxidant enzyme activities that were generally higher than those in the untreated control group. The activities of SOD, POD, CAT, and PPO showed a pattern of initial increase followed by a subsequent decline. This trend may be attributed to pathogen infection, which involves the release of toxins and the action of cell wall-degrading enzymes that cause membrane lipid peroxidation and trigger excessive accumulation of reactive oxygen species (ROS) in plant cells [60]. Under combined pathogen stress and endophyte influence, the signaling role of ROS may be amplified, resulting in a more pronounced induction of antioxidant enzymes [61]. Supporting this, Khan et al. [62] observed significant increases in SOD, POD, and CAT activities in soybean leaves inoculated with *Bipolaris* sp. CSL-1 under salt stress. Similarly, Li et al. [29] reported elevated activities of SOD, POD, CAT, and APX in citrus leaves following inoculation with *Diversispora spurca* (C.M. Pfeiff., C. Walker & Bloss), C. Walker & A. Schüsler, and *Acaulospora scrobiculata* Trappe. Farouk et al. [63] demonstrated that extracts from endophytic fungi isolated from jujube leaves possessed strong antioxidant and antimicrobial activities, with antioxidant effects correlating with enzyme activities such as SOD and CAT, thus providing a resource for natural antioxidant development. These findings align with the present study’s observation that treatment with endophytic fungi significantly enhanced defense enzyme activities. Moreover, Savani et al. [64] reported that co-application of *Trichoderma reesei E.G. Simmons* fermentation broth and *Fusarium oxysporum* spores on banana plants resulted in significant increases in SOD, POD, and CAT activities within 48 h, accompanied by a 30% reduction in malondialdehyde (MDA) content, indicative of decreased membrane damage. Sonia et al. [65] systematically reviewed the interaction between endophytic fungi and pathogens, highlighting that endophytes can prime plant defenses by secreting ROS-scavenging enzymes and signaling molecules to bolster pre-activated resistance. Collectively, these studies corroborate the results of this research, demonstrating the pivotal role of endophytic fungi in modulating antioxidant defenses during pathogen challenges.

In future studies, high-throughput sequencing technology will be employed to analyze the effects of strain DJW5-2-1 on the diversity, community structure, and functionality of rhizosphere microorganisms in rhododendron soil. Transcriptomic and metabolomic analyses will be utilized to dissect the response mechanisms of interaction between *Diaporthe* fungi and their host plants, thereby further exploring the biocontrol and growth-promoting mechanisms of strain DJW5-2-1 in rhododendron species. This biocontrol agent was successful against *C. jiangxiense* (the species investigated), but it also needs to be studied with other species of pathogenic fungi that cause the disease.

## 5. Conclusions

Strain DJW5-2-1 not only secretes a range of cell wall-degrading enzymes that inhibit pathogen growth by compromising their cell walls, but can also reduce the disease incidence of RBS, and exhibits multiple plant growth-promoting traits, enhancing rhododendron seedling development as reflected by increases in plant height, stem diameter, and chlorophyll content. Additionally, this strain boosts plant defense by elevating the activities of key antioxidant enzymes, including SOD, POD, CAT, and PPO, in rhododendron seedlings. Together, these biocontrol and growth-promoting capabilities position strain DJW5-2-1 as a promising candidate for the development of effective biocontrol agents.

## Figures and Tables

**Table 1:** Inhibition rates of five biocontrol fungi against the pathogenic fungus causing RBS.

Strains	XY-4	
	Colony Diameter (mm)	Inhibition Rate (%)
CK	81.82 ± 0.23 a	
DJY1-1	39.79 ± 1.85 b	51.36 ± 2.26 c
DJW5-2	38.25 ± 0.76 b	53.24 ± 0.93 c
DJW5-2-1	29.56 ± 1.25 d	63.88 ± 1.53 a
DJW5-3	34.6 ± 1.16 b	57.72 ± 1.42 b
DJW5-4	32.97 ± 1.39 c	59.70 ± 1.70 b

Note: Different lowercase letters indicate significant differences at *p* < 0.05.

**Table 2:** GenBank accession numbers of strains used for phylogenetic analysis.

Strain	Species Latin Name	Accession Number		
		ITS	TEF	TUB
DJW5-2-1	*Diaporthe phoenicicola*	PV915598	PV948861	PV948860
MFLUCC 24-0368	*Diaporthe phoenicicola*	PQ699356.1	PQ659935.1	PQ659927.1
MFLUCC:24-0367	*Diaporthe phoenicicola*	PQ699364.1	PQ659937.1	PQ659930.1
MFLUCC:24-0366	*Diaporthe phoenicicola*	PQ699363.1	PQ659933.1	PQ659925.1
CBS 111811	*Diaporthe amygdali*	KC343019	KC343745	KC343987
CBS 126680	*Diaporthe amygdali*	KC343023	KC343749	KC343991
CFCC 52586	*Diaporthe kadsurae*	MH121521	MH121563	MH121600
CFCC 52587	*Diaporthe kadsurae*	MH121522	MH121564	MH121601
CFCC 50469	*Diaporthe betulae*	KT732950	KT733016	KT733020
CBS 462.69	*Diaporthe pseudohoenicicola*	KC343184	KC343910	KC344152
CBS 176.77	*Diaporthe pseudohoenicicola*	KC343183	KC343909	KC344151
MFLU 18-2599	*Diaporthe pseudohoenicicola*	MW114352.1	MW192215.1	MW148272.1
NCYU 19-0024	*Diaporthe pseudohoenicicola*	MW114353.1	MW192216.1	MW148273.1
CBS 186.37	*Diaporthe eres*	MH867392.1	KC343810.1	KC344047.1
CBS 102.81	*Diaporthe eres*	KC343074.1	KC343800.1	KC344042.1
CBS 160.32	*Diaporthe vaccinii*	KC343228	KC343954	KC344196
CBS 114015	*Diaporthe ambigua*	KC343010	KC343736	KC343978
CBS 117167	*Diaporthe ambigua*	KC343011	KC343737	KC343979
CBS 161.64	*Diaporthe arecae*	KC343032	KC343758	KC344000
MFLUCC 24-0427	*Diaporthe arecae*	PQ699352.1	PV768975.1	PV753770.1
CBS 535.75	*Diaporthe arecae*	KC343033	KC343759	KC344001
CBS 127271	*Diaporthe novem*	MH864504.1	KC343883.1	KC344125
CBS127270	*Diaporthe novem*	MH864503.1	KC343641.1	KC344124.1
CBS 121124	*Diaporthella corylina*	KC343004	KC343730	KC343972

**Table 3:** Biocontrol efficacy of strain DJW5-2-1 against RBS.

	In Vitro Biocontrol Efficacy		Potted Plant Biocontrol Efficacy	
	Disease Index	Biocontrol Efficacy (%)	Disease Index	Biocontrol Efficacy (%)
CK	79.63 ± 5.23 a		37.17 ± 2.46 a	
DJW5-2-1	40.37 ± 7.11 b	49.67 ± 5.97	18.33 ± 1.18 b	50.61 ± 2.01

Note: Different lowercase letters indicate significant differences at *p* < 0.05.

**Table 4:** Effects of the strain on growth indices of rhododendron seedlings.

Growth Indices	CK	DJW5-2-1	Growth-Promoting Efficiency/%
plant height/cm	20.3 ± 1.28 b	23.4 ± 1.68 a	15.27
root length/cm	4.95 ± 0.73 b	5.62 ± 0.75 ab	13.53
stem diameter/cm	0.24 ± 0.04 b	0.34 ± 0.04 a	41.27
leaf area/cm^2^	2.35 ± 0.38 b	3.16 ± 0.64 a	34.47
chlorophyll content mg/g	2.21 ± 0.05 b	3.44 ± 0.07 a	55.66
aboveground fresh weight/g	1.30 ± 0.07 b	2.37 ± 0.24 a	82.31
aboveground dry weight/g	0.57 ± 0.06 b	1.11 ± 0.08 a	94.73
root fresh weight/g	0.32 ± 0.06 b	1.15 ± 0.04 a	259.37
root dry weight/g	0.14 ± 0.005 b	0.40 ± 0.03 a	185.71

Note: Different lowercase letters indicate significant differences at *p* < 0.05.

## Data Availability

The original contributions presented in this study are included in the article/Supplementary Materials. Further inquiries can be directed to the corresponding author.

## Supplementary Materials

The following supporting information can be downloaded at https://www.mdpi.com/article/10.3390/jof11100728/s1. Table S1: The medium formulations used in this study can be found in the Supplementary Materials.
