# Supplementary material for: Identification of the Antagonistic Fungus Diaporthe phoenicicola Against Rhododendron Brown Spot Disease and Its Disease Control and Plant Growth-Promoting Efficacy
Source: J Fungi (Basel). 2025 Oct 10;11(10):728. doi: 10.3390/jof11100728 (PMC12565191; doi:10.3390/jof11100728)
Supplement: Supplementary file 1 [file jof-11-00728-s001.zip › jof-3855966-supplementary.pdf]

Table S1 Related medium formulations

Media	Chemicals
Potato dextrose agar medium	Potato 200 g, Anhydrous dextrose 20 g, Agar 20 g, H2O 1,000 mL
Potato dextrose medium	Potato 200 g, Anhydrous dextrose 20 g, H2O 1,000 mL
Potato sucrose agar medium	Potato 200 g, Sucrose 20 g, Agar 20 g, H_2O1,000 mL
Carrot agar medium	Carrot 200 g, Glucose 20 g, Agar20 g,H2O 1,000 mL
Cellulase detection medium	Peptone 5 g, Agar 20 g, Congo Red 0.05 g, CMC-Na 1 g, KH_2PO_4 1 g, $MgSO_4·7H_2O$ 0.5 g, $(NH_4)_2SO_4$ 1 g, H2O 1,000 mL
β-Glucanase detection medium	Dextran 5 g, Congo Red 0.05 g, Agar 20 g, KCl 0.5 g, K_2HPO_4 1 g, $MgSO_4·7H_2O$ 0.5 g, $NaNO_3$ 2 g, H2O 1,000 mL
Protease detection medium	Skim Milk Powder 5 g, Agar 20 g, Peptone 10 g, Beef Extract 3 g, NaCl 5 g, H2O 1,000 mL
Amylase detection medium	Soluble Starch 1 g, Agar 2 g, K_2HPO_4 0.03 g, KNO_3 0.1 g, NaCl 0.05 g, $MgSO_4·7H_2O$ 0.1 g, H_2O 1,000 mL
Chitinase medium	Colloidal chitin 10-20 g, KH_2PO_4 1 g, K_2HPO_4 1 g, $MgSO_4·7H_2O$ 0.5 g, NaCl 0.5 g, $CaCl_2$ 0.1 g, Agar 20 g, H_2O 1000 mL
Czapek medium + alfalfa juice	Sucrose 30 g, Agar 20 g, KCl 0.5 g, NaNO3 2 g, K_2HPO_4 1 g, $FeSO_4$ 0.01 g, $MgSO_4·7H_2O$ 0.5 g, H2O ， 200 g alfalfa grass, 500 mL water, boil for 30 minutes， make up with water to 1000 mL
Organic phosphorus medium	Anhydrous dextrose 10 g, Lecithin 5 g, Agar 20 g, $(NH_4)_2SO_4$ 0.5 g, NaCl 0.3 g, K_2SO_4 0.3 g, $MgSO_4·7H_2O$ 0.3 g, $MnSO_4$ 0.03 g, $FeSO_4$0.03 g, H2O 1,000 mL
Nitrogen-free medium	Mannitol 10 g, Agar 20 g, KH_2PO_4 0.2 g, NaCl 0.2 g, $CaSO_4$ 0.1 g, $CaCO_3$ 5 g, $MgSO_4·7H_2O$ 0.2 g, H_2O 1,000 mL
Siderophore detection medium	Sucrose 10 g, Peptone 5 g, Yeast Extract 1 g, Agar 20 g, KH_2PO_4 0.2 g, $MgSO_4·7H_2O$ 0.2 g, H2O 1,000 mL
Aleksandrov medium （Potassium-solubilizing medium）	Sucrose 5 g, Agar 20 g, Bromothymol Blue 0.1 g, Potassium Feldspar Powder 1 g, Na_2HPO_4 2 g, $MgSO_4·7H_2O$ 0.5 g, $CaCO_3$ 0.1 g, $FeCl_3$0.05 g, H_2O 1,000 mL
